# Genomic analysis for cattle breeding improvement, progress and future perspectives in Peru: a review

**DOI:** 10.1080/10495398.2025.2547344

**Published:** 2025-08-31

**Authors:** Deyanira Figueroa, Yolanda Romero, Lizeth A. Heredia-Vilchez, Carlos Poemape, Wigoberto Alvarado, Carlos Quilcate

**Affiliations:** a Dirección de Investigación y Desarrollo Tecnológico – DIDET, Instituto Nacional de Innovación Agraria, Lima, Perú; b Instituto de Investigación en Ganadería y Biotecnología, Universidad Nacional Toribio Rodríguez de Mendoza, Amazonas, Perú; c Unidad de Imagen Institucional, Instituto Nacional de Innovación Agraria, Lima, Perú; d Facultad de Ingeniería Zootecnista, Agronegocios y Biotecnología, Universidad Nacional Toribio Rodríguez de Mendoza de Amazonas, Amazonas, Perú

**Keywords:** Genomics, cattle, review, Peru

## Abstract

Genomics offers a promising solution by enabling precise cattle selection and breeding to boost productivity and sustainability. In Peru, livestock plays a crucial role in the economy and food security. Despite its importance, the sector faces significant challenges, including poor pasture quality, limited conservation practices, a shortage of trained professionals, minimal use of genomic tools, and an incomplete understanding of the genetic potential of both native and introduced breeds. Since the 1940s, Peru has advanced in genetic improvement through artificial insemination, improved semen preservation, the establishment of a National Semen Bank, and the introduction of new breeds. Key developments have included embryo transfer, in vitro fertilization, and pioneering cloning efforts. Future perspectives for livestock genomics in Peru involve expanding bioinformatics capacity, improving genomic infrastructure, and integrating genomic selection into national breeding strategies. This review discusses the history, current status, challenges, and future perspectives of livestock genomics in Peru.

## Introduction

Livestock production is a crucial pillar of Peru’s economy and food security in Peru, accounting for 6% of the Gross Domestic Product (GDP) and employing 24% of the Economically Active Population (EAP), or 4 million jobs nationwide.^1^ This sector is made up of approximately 1,764,660 rural households, of which 486,829 are cattle breeders, mainly small farmers and peasant communities that own Creole cattle and their crossbreeds. Peru currently has a cattle population of approximately 5,101,895 head, which produces 1,115,045 tons of milk and 135,854 tons of meat annually.^2^ The cattle sector is composed of around 1,764,660 rural households, of which 486,829 are cattle breeders, primarily small-scale farmers and peasant communities owning Creole and crossbred cattle.^3^ Approximately 70% of the national herd is managed under smallholder systems, with most producers owning fewer than 10 animals.^2^^,^^4^ In contrast, commercial and large-scale producers represent a smaller proportion of the total, but manage improved breeds and utilize more intensive systems, especially in coastal dairy basins.

Despite its importance, the sector struggles with several critical issues to achieve sustainable production that meets the growing demand for meat and milk, while minimizing its environmental and social impacts. The seasonality of production, driven by fluctuating weather patterns, leads to inconsistent supply and price volatility, which affects both producers and consumers.^5^ Additionally, the quality and availability of pasture and forage are insufficient, with many regions experiencing overgrazing and soil degradation, further impacting the sustainability of feed sources. Conservation techniques are underdeveloped, resulting in poor management of forage and crop residues that could otherwise enhance livestock nutrition.^6^

The genetic quality of the cattle population remains a major challenge. With around 90% of cattle being Creole and crossbreeds,^4^ the productivity is generally lower compared to improved breeds, particularly imported breeds such as Holstein, Brown Swiss, Jersey, Simmental, and Brahman, which have been introduced for their higher milk or meat yields. This is compounded by low reproductive efficiency, high mortality rates of young animals, and the high cost of intensive production systems, which affects profitability.^2^ Moreover, the scarcity and cost of high-quality breeding stock limit opportunities for genetic improvement. There is also a noticeable delay in adopting advanced technologies and practices, hindering overall progress.

Bovine genetic improvement, through the application of genomics, is a key tool to overcome these challenges, increasing productivity and efficiency, and improving the quality of derived products. Genomics enables a more precise approach to cattle selection and breeding, facilitating the identification of desirable genetic traits that can improve production in a sustainable manner and reduce negative environmental impact.^7^ Additionally, advancements in feed technology and conservation methods could enhance the quality and availability of nutrition, while improved management practices can address environmental impacts.

Policy support is critical to integrating these advanced technologies into the sector. Investment in research, infrastructure, and education is essential to foster innovation and ensure that small-scale farmers can benefit from new developments. Strategic partnerships between government agencies, research institutions, and industry stakeholders will be crititcal to achieving sustainable growth and strengthening the sector’s resilience to future challenges.

This review provides a comprehensive analysis of the current state of genomic research in cattle breeding in Peru, assesses the progress made in leveraging genomic tools for improving livestock genetics, and explores future perspectives and potential advances in this field. This review aims to identify key achievements, challenges, and opportunities in the application of genomic technologies to improve cattle breeding practices, and to propose strategies for integrating these advances into Peru’s agricultural sector to achieve sustainable and improved livestock production.

## History of Peruvian cattle genetic improvement

**Figure 1.**
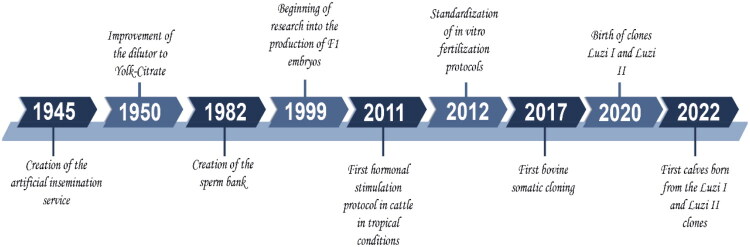
Time-line of the history events of the Peruvian cattle genetic improvement.

Genetic improvement in Peruvian cattle breeding has progressed considerably since the 1940s (Figure 1), with the advent of artificial insemination as a key milestone. In 1943, Dr. Mackenzie performed the first artificial inseminations in sheep at the LAIVE farm, located in Huancayo, which marked the beginning of the application of biotechnology in Peruvian livestock. In 1945, the artificial insemination service for cattle was officially established in Peru with the importation of Holstein bulls from Canada. This service underwent a significant improvement in 1950 with the implementation of the Yema-Citrate dilutor, which optimized semen preservation and increased the efficiency of the artificial insemination process.^8^^,^^9^

In 1982, the National Semen Bank was created, a key institution in the field of genetic improvement in Peru., was established. This bank has played a crucial role in selecting sires from various regions of the country and distributing millions of semen straws, which has significantly contributed to the increase in the genetic quality of Peruvian cattle.^10^^,^^11^

The 1970s represented a crucial period for genetic improvement in Peruvian cattle breeding, with the introduction of the Simmental and Fleckvieh breeds in the highlands through an agreement with the German Technical Cooperation. These breeds, designed for the dual purpose of milk and meat production, demonstrated remarkable adaptability to the harsh conditions of the Andean region.^12^ In 1999, the Peruvian Ministry of Agriculture intensified genetic improvement efforts by importing Fleckvieh embryos from Germany. These embryos were implanted in cows from the Universidad Nacional Agraria La Molina (UNALM) and the Sociedad Agrícola de Interés Social (SAIS) Túpac Amaru, which was a significant milestone in the expansion of this genetics in the country.^13^

The development of advanced biotechnologies has played a fundamental role in the genetic improvement of cattle breeding in Peru. In 1999, at the Artificial Insemination and Embryo Transfer Laboratory of the Calzada Livestock Farm, research was initiated to produce F1 bovine embryos (Gir Lechero x Holstein and Gir Lechero x Brown Swiss), with the objective of optimizing productivity in both milk and meat. This effort was expanded in 2003 with the implementation of in vitro embryo production (PIVE), although still in its early stages, reaching success rates below 30%.^14^

In 2011, important advances were recorded in bovine embryo production under tropical conditions in Peru, using a hormonal stimulation protocol that allowed achieving a pregnancy rate of 40.9% in recipient cows. This result confirmed the efficacy of embryo transfer as a key tool in the genetic improvement of dairy cattle in the country. The following year, in 2012, in vitro fertilization protocols were standardized in the Reproductive Biotechnology Laboratory of the EEA Canaán, obtaining up to 40% of viable embryos, which represented a significant advance in the development of adaptive technologies (INIA, 2012).

In 2014, the National Institute for Agrarian Innovation (INIA) inaugurated the first Animal Reproductive Biotechnology Laboratory in Junín, which represented a significant boost in the production and transfer of embryos with high genetic quality. This infrastructure has considerably shortened the time needed to obtain purebred animals, in addition to improving accessibility to breeders of high genetic value for small producers in the region (INIA, 2014).

The Universidad Nacional Toribio Rodríguez de Mendoza (UNTRM) has stood out as a leader in bovine cloning in Peru. In 2015, UNTRM conducted the first cloning experiment using bipartition in the country, followed in 2016 by the first bovine cloning employing the Handmade Cloning technique.^15^ In 2017, the institution achieved a significant milestone with the first bovine somatic cloning in Peru, which resulted in the birth of the Alma C-I clone.^15^ This progress continued in 2020 with the birth of clones Luzi I and Luzi II,^16^ which were subsequently inseminated in 2022 with semen of high genetic value in 2022, resulting in the birth of two calves, representing a significant progress in the genetic improvement of Fleckvieh-Simmental cattle in the country.^17^

## Evolution of genomic technologies in animal husbandry

### Introduction of genomic technologies in the last decade

Rapid advances inmolecular genetics have enabled the direct quantification and detailed study of genomic diversity, eliminating the need for statistical inference based on genealogical data, even in populations without genealogical records.^18^^,^^19^ Currently, molecular genetic markers are among the most effective tools for genomic analysis, as they allow the study of the association between heritable traits and underlying genomic variation. This capability is fundamental not only for analyzing genetic diversity within a population but also for investigating genetic relationships between different populations. Before the introduction of DNA analysis, biochemical polymorphic traits were traditionally used to study genetic differences within and between populations and to estimate genetic divergence.^20^ During the 1970s, numerous studies analyzed genetic variability between livestock populations using blood groups and allozyme systems.^21^ These studies became indispensable for optimizing selection strategies in farm animals, as some polymorphic alleles can be associated or linked to economically important traits.

The progress of genomics and its applications in livestock farming has led to significant improvements in the biological prediction of various genetic traits. Leading dairy-producing countries, such as Canada, Ireland, Australia, the United States, France, the United Kingdom, the Netherlands, New Zealand, Germany, and the Scandinavian countries have integrated genomic evaluations into their breeding programs, resulting in important changes in the global dairy industry.^22^ In addition to the dairy sector, genomic technologies have also significantly advanced beef cattle breeding. Breeds such as Angus, Hereford, Simmental, and Brahman have benefited from the identification of genomic regions associated with growth, feed efficiency, meat quality, and adaptability to different environments.^23^^,^^24^ These advances highlight the broad applicability of genomics across both dairy and beef cattle sectors. The evolution of genomics shows how this technology has become an essential tool, moving from genome sequencing to approaches such as genome-wide association studies (GWAS), whole-genome prediction (WGP), and the selection of complex traits through genomics.^25^ In the 1990s, initial genetic evaluations using microsatellite markers successfully identified quantitative trait loci (QTLs), which were associated with the variation of quantitative traits in the phenotypes of a population.^26^

To advance these technologies in the cattle industry, the first genome sequencing studies of the cattle species (Bos taurus) were conducted in Hereford cattle.^27^ Later, the Holstein breed genome was sequenced and used as a reference to compare genomic variation with the Hereford breed.^28^ In bovine species, approximately 22,000 genes have been identified through sequencing analyses, providing a fundamental dataset for future applications in dairy cattle selection programs.^27^^,^^28^ In Bos indicus, the genome has also been sequenced in the dairy breeds Gyr, Girolando, and Guzerat.^29^ The first commercial genotyping chip, containing about 54,000 single nucleotide polymorphisms (SNPs), was introduced in 2007, allowing genomic evaluations in different dairy breeds such as Holstein, Jersey, and Brown Swiss in the United States since 2009, and later in the Ayrshire and Guernsey breeds in 2013 and 2016, respectively.^7^

### Next-generation sequencing and SNP genotyping

The availability of complete genome sequences has facilitated the use of highly efficient genetic markers (10,000 to 1,000,000 SNPs), significantly improving the accuracy of trait prediction. This technology relies on high-capacity genotyping platforms, such as DNA arrays or SNP chips.^25^ Additionally, technologies such as gene expression profiling using DNA microarrays (MGEP) have become widely used in functional genomics and transcriptomics studies. It is expected that, in the near future, genomics and transcriptomics currently based on SNP chips or microarrays will be replaced by next-generation sequencing (NGS) technologies, combined with statistical biology and bioinformatics.^30^

The advancement of automatic and semi-automatic techniques for detecting DNA polymorphisms has replaced the use of protein polymorphisms with DNA polymorphisms as the preferred markers in genetic diversity studies.^31^ Currently, it is possible to estimate genetic diversity parameters directly from DNA, providing accurate results on genetic variation both among and within breeds, populations, and individuals. Since the 1990s, microsatellite molecular markers have been successfully used in paternity testing, individual identification, breed assignment in domestic animals, and genetic diversity characterization studies.^32–36^ Microsatellite marker maps were developed to estimate the additive genetic effects of these markers and were routinely used to detect quantitative trait loci (QTL) in most livestock species. This resulted in significant advances in genetic diversity analyses.^37–40^

In the last decade, contemporary genetic analysis has emerged thanks to high-throughput sequencing, offering genomic information such as single nucleotide polymorphisms (SNP) and whole-genome sequencing (WGS). SNPs, which are single mutations in the genome sequence present in both coding and non-coding regions, facilitate the analysis of both neutral and functional genetic variation.^41^ The availability of automated techniques, their low cost, and their high prevalence in the genomes of domestic animals have facilitated their adoption in livestock genetic studies. Currently, high-performance commercial SNP arrays are available for most livestock species, targeting genetic variants widely distributed throughout the genome. It is important to note that these arrays are mainly designed using commercial breeds, which may introduce a validation bias concerning local breeds.^42^

## Omics technologies in genetic improvement

The integration of omics technologies, such as genomics, metagenomics, metabolomics, proteomics, transcriptomics, epigenomics, and translatomics, has enabled the rapid and efficient identification of subtle phenotypic variations, dietary responses, and innate predispositions in animals.^43^^,^^44^ The implementation of these technologies in animal selection and breeding programs allows for precise estimation of breeding values for early selection, reduces the generation interval, and increases the rate of genetic gain. Over the past two decades, these methodologies have revolutionized genetic selection in breeding animals.

For instance, in 2007, the first high-density genetic marker panel in cattle was released, containing 54,001 single nucleotide polymorphisms (SNPs), facilitating genome-wide association studies (GWAS) and demonstrating the association between SNPs and quantitative trait loci (QTLs).^45^ Additionally, whole-genome sequencing (WGS) has been fundamental in identifying molecular signatures in selection and breeding.^27^^,^^46^ Molecular databases, such as those from NCBI, EMBL, and DDBJ, are used to analyze genomic diversity and the molecular and physiological basis of traits of economic interest.^47^^,^^48^ Furthermore, omics technologies help identify and prioritize functional SNPs, thereby improving the accuracy of genetic selection.^49^ The combination of multi-omics data with bioinformatics and computational biology tools has given rise to high-dimensional biology (HBD), which allows the simultaneous analysis of genetic variation in genes, transcripts, proteins, and metabolites.^50^^,^^51^

In the context of Peruvian cattle breeding, recent studies have begun to apply high-throughput genomic technologies such as SNP genotyping and next-generation sequencing (NGS).^52^ published the draft genome of a Peruvian Creole bull, which included both nuclear and mitochondrial sequencing using Illumina platforms, marking one of the first implementations of NGS in local breeds. Additionally,^53^ used high-density SNP panels (BovineHD and Bovine100K) to assess population structure and genetic differentiation between commercial (Brahman, Braunvieh, Fleckvieh, Gyr) and Creole cattle, providing actionable data for genetic improvement strategies. These studies highlight the progressive incorporation of advanced genomic tools in Peru and underscore the potential of NGS and SNP technologies to enhance selection accuracy and preserve local genetic resources in future breeding programs.

## Exploring the genome and microbiome: Integration of omics technologies in cattle

Advances in bovine genomics began in 2009 with the publication of the first bovine genome of the Hereford breed, marking a turning point in cattle genetic research. This genome, with over 90% of its assembly correctly assigned to chromosomes and an estimated size of 2.87 Gb, demonstrated remarkable accuracy and completeness. In 2017, the reference genome for beef cattle was updated to its fourth version (ARS-UCD1.2), integrating Illumina and PacBio sequencing technologies, resulting in greater continuity and more accurate gene annotation. Currently, the ARS-UCD2.0 version offers complementary sequencing data and more comprehensive gene annotation, providing more accurate and continuous genomic information for cattle.

The sequencing and assembly of the complete genome of Peruvian Creole cattle revealed an estimated genome size of 2.58 Gb, with a sequencing coverage of approximately 47.44X.^52^ Analysis of the mitochondrial genome of this breed provided additional insights into its phylogeny, showing similarities with other species of the genus Bos and suggesting a possible relationship with native African breeds.^54^ These findings underscore the importance of continuing to explore the genetic diversity of these local populations. Complementary analysis of the mitochondrial genome of the bull “Pumpo,” a prominent specimen from the Peruvian genetic nucleus, revealed a genome of 16,341 bp, comparable in size and organization to other members of the subfamily Bovinae, such as Bos taurus and B. indicus.^55^

The intestinal microbiota plays a crucial role in the health and well-being of farm animals, influencing immune response, behavior, and stress response.^56^ The diversity and composition of the intestinal microbiota are recognized as key indicators of these animals’ health status, and fecal microbiota transplantation (FMT) has been proposed as a promising strategy to improve productivity, health, and well-being. In particular, the relationship between the microbiota-gut-brain axis and the stress response has been studied,^57^ suggesting that microbiota manipulation could offer an effective pathway to mitigate the negative effects of stress in farm animals.^58^

A recent analysis of the intestinal microbiota in a genetic nucleus of cattle revealed that age and sex significantly impact microbial diversity and composition. Older animals had greater bacterial diversity and abundance, while sex had a significant influence on the structure of the microbial community. An increase in the presence of butyrate-producing bacteria, which are essential for intestinal health and inflammation reduction, was observed. Additionally, variations in the microbiota were correlated with changes in blood parameters, underscoring the connection between microbial diversity and overall cattle health.^55^ These findings suggest that future strategies could focus on microbiota manipulation to optimize cattle health and performance, through the development of specialized diets and probiotics tailored to different ages and sexes.

## Current status of cattle raising in Peru

The livestock sector in Peru constitutes an essential component of the rural economy, contributing 41% of the agricultural gross domestic product (GDP).^59^ Despite its importance, livestock production in Peru faces significant challenges that limit its competitiveness and its ability to take advantage of opportunities at the global level. It is estimated that more than 70% of rural households depend on this activity, which remains vulnerable due to its limited profitability and competitiveness.^60^

Livestock production is a central activity in rural Peru and plays a key role in local economies through the commercialization of raw milk, artisanal dairy products and live cattle. Around 35% of national raw milk production is directed to the artisanal industry and self-consumption, with fresh cheese as the main derivative.^60^

The structure of cattle ranching in Peru is divided into three main categories: commercial cattle ranching, small and medium cattle ranching, and subsistence cattle ranching. Commercial cattle ranching, which is concentrated on the coast, is characterized by the use of advanced technologies and specialized breeds, representing approximately 9% of the total cattle population. In contrast, small and medium cattle ranching, found in the coast, highlands and jungle, uses medium to low sophistication technologies. Finally, subsistence cattle ranching, which constitutes 57% of the cattle population, uses low-technology production techniques and is mainly oriented toward subsistence consumption.^61^

**Figure 2.**
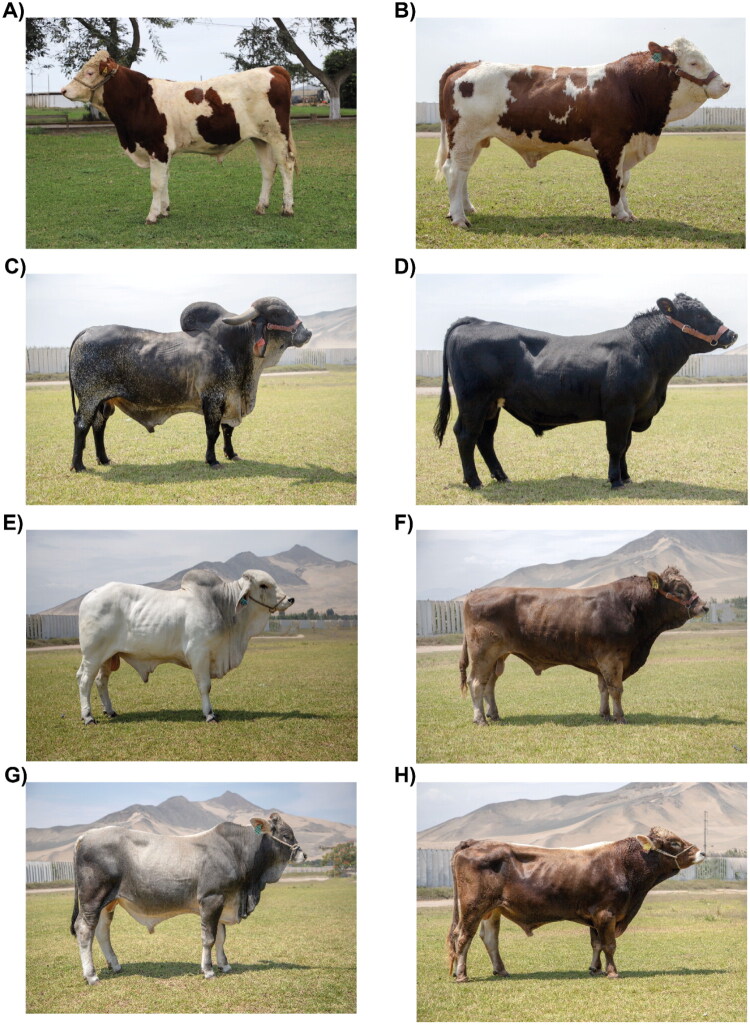
Specialized-breed bulls of the Central genetic nucleus, montbeliarde (a), Simmental (B), Gyr (D), Angus (D), Brahman (E), Braunvieh (F), F1 Simmental x Brahman (G) and Brown Swiss (H).

The Peruvian government has established a specialized herd called the “Central Genetic Nucleus – EEA Donoso” with Brahman, Braunvieh, Gyr, and Simmental cattle breeds to promote research in reproductive technologies. This initiative was designed to improve techniques such as artificial insemination and embryo transfer, aiming to improve livestock breeding efficiency and genetic quality. The project was part of a broader effort to modernize and innovate agricultural practices in Peru, ensuring better productivity and sustainability of the country’s cattle industry.^62^ Nowadays, the Central Genetic Nucleus have specialized-breed bulls such as Montbeliarde, Simmental, Gyr, Angus, Brahman, Braunvieh, F1 Simmental x Brahman and Brown Swiss (Figure 2).

## Genetic variability

Cattle raising in Peru is characterized by the predominance of Creole cattle, which represent approximately 65% of the country’s total cattle population.^4^ These cattle, direct descendants of cattle brought from the Iberian Peninsula in the 15th century, have developed a remarkable adaptation to local conditions, especially in the Andean regions. Creoles are highly valued for their ability to survive in environments with limited water resources and difficult terrain, making them a strategic option for livestock production under extreme climatic conditions.^63^ In addition, these breeds have shown resistance to disease and play a crucial role in the conservation of local biodiversity and in the production of high-quality meat.

Recent research on ovarian follicular dynamics in Creole cows under grazing conditions in the high Andean zone of Peru highlights their ability to maintain a stable reproductive cyclicity, independent of seasonal variations in nutrient availability. This suggests the presence of adaptive physiological mechanisms that are crucial for their conservation and genetic improvement in high Andean livestock, which is fundamental for the implementation of reproductive biotechnologies.^64^

On the other hand, fasciolosis, caused by Fasciola hepatica, is one of the most important zoonotic diseases affecting cattle in the Andean region, with a prevalence of 55.72% in slaughterhouses in Huancayo. This disease not only causes significant direct economic losses due to the condemnation of infected livers, but also indirect losses due to the reduction in body weight of affected animals.^65^

Analysis of the Y chromosome in Peruvian Creole cattle has revealed low genetic diversity, with a clear influence of the European Y1 and Y2 haplogroups, suggesting a predominantly Iberian origin. An African influence has also been identified, although no evidence of Indic paternal lines was found.^66^ This unique genetic diversity is essential for the development of breeding programs and the conservation of genetic resources in the country.

Genetic diversity and population structure analysis comparing Braunvieh, Simmental, Brahman and Gyr with Creole cattle from Arequipa, a southern Peruvian region, grouped the Creole into a single cluster, suggesting the importance of their distinctive genetics,^53^ and the same analysis between four Peruvian regions (Cusco, Apurimac, Ayacucho and Puno) revealed that Puno Creole cattle differed from the other regions Creoles.^67^

Despite the importance of these creole breeds, they have been underestimated in terms of genetic research and breeding programs, which limits their potential to contribute to more sustainable livestock production adapted to local conditions. This situation highlights the need for more detailed genetic and phenotypic studies to maximize the use of these resources to improve livestock production in Peru.^68^

## Genomics applications in Peruvian cattle breeding

Genomic resources are crucial for bovine genetic improvement in Peru, as they allow the selection of animals with optimal traits such as higher productivity and disease resistance. This optimizes production efficiency and contributes to sustainability by reducing inputs and better adapting to local conditions and climate change, thus promoting stronger and more profitable livestock farming.

Advanced sequencing of bovine genomes, facilitated by next-generation technologies, began with the sequencing of the first genome in 2009.^69^ Additionally, the development of commercial SNP chips has improved the understanding of cattle genomics and has led to the creation of SNP chips of different densities.^26^^,^^45^^,^^70^ These advances are being used to study genetic diversity, detect signatures of selection, identify important genetic variants that can be associated with production traits, and improve livestock production through genomic selection.

In Peru, the development of genomic resources for cattle is progressing thanks to the work of institutions and universities, however, these are scarce (Tables 1 and 2). In 2022, the mitochondrial genome of a Creole bull from the Arequipa region was published and the phylogenetic analysis grouped it with individuals from Africa;^54^ in the same year, the draft of the complete genome of this individual was published.^52^ The latest efforts were made this year, with the publication of the mitochondrial genome of a bull that is part of a central genetic nucleus.^55^ In addition, the country participates in international networks and collaborates with global organizations to access advanced genomic resources such as the Bovine Pangenome Consortium.^71^

**Table 1.** Reference genomes of animals sequenced in Peru.

Individual	Release year	Sequencing center	Accession (bioproject)
Creole cattle	2021	Instituto Nacional de Innovación Agraria	PRJNA763011
Simmental (from genetic nucleus)	2024	Instituto Nacional de Innovación Agraria	PRJNA1097623

**Table 2.** Synopsis of genomic applications in livestock improvement in Peru.

Genomic application	Description	Impact
Genomic selection	Identification of animals with superior productive traits and disease resistance.	Increased productivity and sustainability in livestock farming.
Genome Sequencing	Use of advanced technologies to sequence bovine genomes and enhance genetic knowledge.	Better understanding of the bovine genome and its application in genetic improvement.
Genetic diversity analysis	Study of genetic diversity in cattle breeds using SNP panels.	Conservation and enhancement of local breeds with a focus on genetic diversity.
Time-Lapse technology (Geri^®^)	Continuous evaluation of embryonic development to optimize viable embryo selection.	Improvement in success rates of assisted reproduction programs.
Computerized Semen Analysis (CASA)	Automated evaluation of semen quality to improve artificial insemination efficiency.	Ensures better quality of semen used in reproduction, optimizing costs.
Disease resistance	Identification of genes related to resistance against diseases such as fascioliasis.	Reduction of economic losses and improvement in animal health.
Optimization of milk production	Use of recombinant bovine somatotropin (rbST) to increase milk production efficiently.	Increase in dairy production profitability without compromising animal health.
Lactation curve modeling	Application of mathematical models to analyze milk production patterns and optimize herd management.	Optimization of milk production throughout the lactation cycle.

On the other hand, a study performed a diversity analysis among Brahman, Braunvieh, Gyr, Fleckvieh and Peruvian Creole using two SNP panels (BovineHD and Bovine100K), making the data available to other users.^53^ Although significant genomic resources have been developed and accumulated for bovine research and genetic improvement, the technology used to date has not incorporated the most advanced sequencing techniques, such as those offered by the PacBio platform. This next-generation sequencing technology provides much longer and more accurate DNA reads compared to traditional technologies, allowing for better resolution of genome structure and more detailed identification of genetic variants and regions of interest.^72^

In the reproduction field, the Geri^®^ time-lapse technology has emerged as an essential tool forin vitro production (IVP) of embryos, particularly in pigs and cattle, allowing for continuous and detailed observation of embryonic development.^73^^,^^74^ This system captures images at five-minute intervals, providing a precise assessment of kinetic events during the early stages of development. The time required to reach the 2-cell stage was found to be a significant predictor of blastocyst formation, suggesting that morphokinetic analysis through time-lapse not only optimizes embryo selection, but also improves incubator space management and facilitates the rapid evaluation of new culture media formulations.^75^^,^^76^

**Figure 3.**
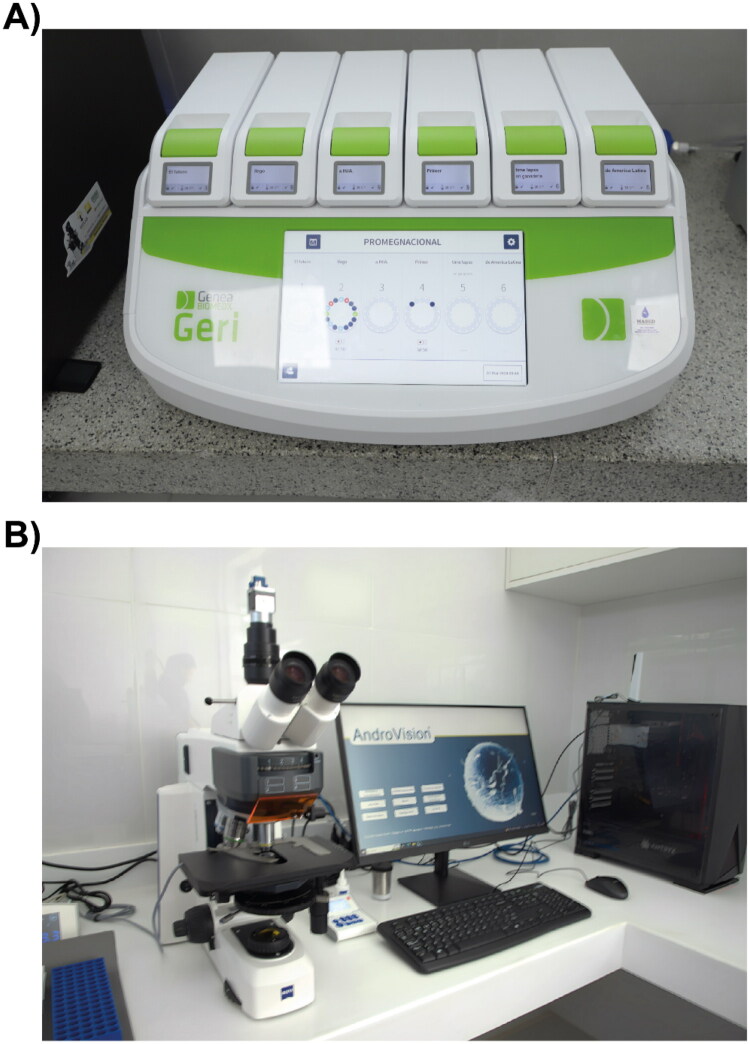
Geri^®^ (A) and CASA (B) system at the laboratory of the Central genetic nucleus (Donoso Experimental Station).

In Peru, the implementation of the Geri^®^ system (Figure 3A) at the Central Genetic Nucleus of the Donoso Experimental Station of the National Institute of Agrarian Innovation (INIA) represents a significant advance in cattle genomic selection. This technology, originally adapted for human embryos, has been successfully applied to in animal production, allowing continuous and precise monitoring of embryonic development.^77^ Its application is expected to increase efficiency in identifying the best candidates for transfer, thereby optimizing results in genetic improvement programs. The Geri^®^ system provides an advanced tool for real-time morphokinetic evaluation, with the potential to have a significant impact on the country’s livestock production (PI CUI 2432072).

Additionally, the computer-assisted sperm analysis (CASA) system (Figure 3B) has revolutionized semen evaluation in livestock production, providing a crucial tool for improving the precision and objectivity in selecting high-quality semen for artificial insemination.^78^ Unlike traditional methods, CASA offers a detailed analysis of sperm motility, morphology, and concentration, which are fundamental factors in determining the fertilizing capacity of spermatozoa.^78^ The implementation of CASA in semen production centers optimizes sperm selection, ensuring that only those with the best characteristics are used, reducing the need for additional doses, and improving the overall efficiency of the reproductive process. Furthermore, CASA provides quantifiable and reproducible data, facilitating the comparison of results between laboratories and breeding centers, thereby strengthening the scientific basis for decision-making in livestock management.^79^

The INIA under MIDAGRI has implemented the first Computerized Bovine Semen Analysis System (CASA) in Huaral, allowing for an automated and precise evaluation of bovine semen. This technology aims to improve the genetic quality of cattle in Peru, promoting the production of offspring with higher productivity in meat and milk, thereby benefiting small and medium-sized farmers in the country.^80^

In the Amazonas region of Peru, a significant prevalence of fascioliasis has been documented in cattle, reaching 90.13%. This disease, caused by the parasite Fasciola hepatica, represents a considerable challenge to productivity, affecting meat and milk production and leading to economic losses due to the condemnation of infected livers. It is noteworthy that there are differences in susceptibility to infection between breeds, with the Brown Swiss breed being more susceptible and Creole cattle showing greater resistance. These results highlight the importance of using genomic tools to improve disease resistance, a crucial strategy for the sustainability and efficiency of livestock production in the region.^81^

The integration of bovine genomics in the improvement of Creole breeds has the potential to increase the sustainability and efficiency of livestock production in Peru. Through the selection of desirable traits, such as resource use efficiency and disease resistance, it is possible to optimize productivity without compromising ecosystem health. In addition, genomics can identify and preserve genes associated with the nutritional quality of meat, such as omega-3 fatty acid content, which is highly valued in organic and high-quality meat markets.^63^

The application of recombinant bovine somatotropin (rbST) has been demonstrated as an effective biotechnological tool to increase milk production without compromising cow body condition. This is particularly relevant in the Peruvian context, where increased milk production can translate into higher income for farmers, improving profitability in intensive production systems. However, it is crucial to consider the variability in animal response, influenced by factors such as management, feeding and climatic conditions, typical of tropical and subtropical regions. Research tailored to local conditions is essential to fully assess the impact of rbST and other biotechnologies on the sustainability of livestock production. The integration of these technologies must be carefully planned to ensure their alignment with sustainable and ethical practices in livestock farming, maximizing benefits and minimizing risks to animal welfare and the environment.^82^

Finally, the modeling of lactation curves is presented as a valuable tool in the evaluation of milk production. A study conducted in Holstein cows in Peru used nonlinear mixed models, such as those of Wood and Wilmink, to analyze the lactation curve in an intensive production system. These models made it possible to identify production patterns and determine peak lactation and persistency, which is fundamental to improve efficiency and profitability in dairy production. The application of these models, together with genomic tools, can optimize the selection and management of high productivity cows, promoting more sustainable and profitable practices in dairy farming in the country.^83^

## Culture and adoption of new technologies

In a study conducted in the district of Moyobamba, identified three groups of livestock producers with different characteristics were identified, underscoring the importance of education and the adoption of technological innovations. Producers with higher educational levels, especially those with university education, showed a greater willingness to adopt new technologies, including genomic applications in livestock farming. The assessment of sustainability on these farms revealed significant challenges related to soil quality, pasture health and livestock quality, which are fundamental aspects of environmental and economic sustainability. The implementation of silvopastoral systems was highlighted as an effective strategy to improve these conditions, promoting a more sustainable and efficient management of natural resources. The active participation of producers and the strengthening of their capacities in infrastructure and resource management are essential to ensure the success of these strategies, highlighting the need for training programs and technical support.^84^

A study in Cajamarca, Peru, on antibiotic use on small dairy farms revealed that lack of education and traditional practices can negatively affect proper animal health management. Veterinarians and feed vendors identified challenges such as limited availability of appropriate drugs and competition among prescribers as contributing to inappropriate antibiotic use. This problem reflects a broader challenge in the adoption of modern technologies, such as genomics, in Peruvian livestock farming. Improving farmer education and training is crucial to promote sustainable and effective livestock management practices, thus ensuring successful implementation of new technologies.^85^

In addition, in Langui, Peru, it was observed how global market pressures and climate change are leading to reduced crop diversity and increased production of livestock fodder in response to demand from transnational dairy companies. This shift represents a significant challenge for the adoption of advanced genomic technologies, as rural communities often prioritize practices that secure immediate income over long-term investments that could preserve agrobiodiversity and improve productivity in a sustainable manner. Urban migration and climate change also accelerate the loss of traditional agricultural knowledge and practices, underscoring the need for educational strategies and supportive policies that facilitate a balanced transition to advanced technologies while preserving the ecological resilience and cultural identity of local communities.^86^

The Dairy Production Service, initiated in 1950 by the Universidad Nacional Agraria La Molina in coordination with the Ministry of Agriculture and the Lima Basin Cattlemen’s Association, has been fundamental in evaluating the productivity characteristics of dairy cows. Beginning in 1965, “Control Lechero” began electronic data processing, expanding to eleven basins and evaluating more than 2,083 barns nationwide. However, the lack of productivity data and limited awareness of the benefits of bovine genomics suggest the need for more training in genomic technology management.^87^

In addition, national genetic improvement programs include methods such as artificial insemination and embryo transfer, promoted by the National Institute for Agrarian Innovation (INIA) and the Ministry of Agrarian Development and Irrigation (MIDAGRI). These technologies, although effective, require greater understanding and proper management by producers to maximize their impact.^88^ Training is essential for farmers to understand and adopt these technologies, including practices such as record management and economic mating.

## Challenges in the implementation of genomics in Peru

Foot-and-mouth disease (FMD) represents a considerable threat to the livestock industry in Peru, with potential economic and health repercussions. A multidisciplinary approach has made it possible to assess the risks associated with possible FMD epidemics, using methods such as spatial stochastic modeling to simulate disease transmission and social network analysis to understand farm-to-farm contact patterns. These studies indicate that most infections occur through local transmission, highlighting the importance of livestock insurance systems to mitigate the costs associated with FMD outbreaks. These systems would not only compensate affected farmers financially, but also encourage sound management practices to reduce the risk of future outbreaks, especially in areas with high livestock densities.^89^

Livestock systems in the Peruvian Andes are multifunctional, providing products such as milk and meat, as well as essential services such as organic fertilizer and traction power.^90^ A Life Cycle Assessment (LCA) study identified enteric fermentation as the main source of greenhouse gas emissions and highlighted pasture management as a determining factor in acidification and eutrophication. It is critical that economic assessments adequately reflect the importance of these functions to rural communities, suggesting a more holistic approach to environmental impact assessment.^91^

The implementation of bovine genomics in Peru has the potential to improve the productivity and sustainability of livestock production, especially in high mountain livestock micro-watersheds where water and soil quality degradation is a growing problem. Genomic technology can facilitate the selection of livestock adapted to adverse conditions, such as eroded soils and limited water resources, improving the resilience and sustainability of production.^83^^,^^92^ However, limited infrastructure and the need for human resource training are significant challenges. Creole breeds, known for their adaptability, could benefit greatly from these applications, allowing for more precise genetic selection that promotes resilience. It is crucial to develop public policies that support research and adoption of these technologies, in addition to fostering international collaborations that provide the necessary technical and financial support.^63^

The seroprevalence of reproductive and infectious diseases in cattle in Madre de Dios, Peru, such as bluetongue (BTV), enzootic bovine leukosis (BLV) and bovine herpesvirus type 1 (BHV-1), reveals significant challenges. Factors such as interaction with vectors and natural reservoirs of pathogens, exacerbated by climate change, underscore the need to integrate disease management strategies into breeding programs, prioritizing genetic resistance. The identification of endemic diseases and their impact on production highlight the importance of implementing robust biosecurity, vaccination and constant monitoring programs to ensure the sustainability and profitability of livestock production in tropical and subtropical regions of Peru.^93^

## Challenges in cattle breeding and genomics adoption in Peru

### Limited infrastructure and resources

Due to fragmented ownership, cattle management in Peru is done through small herds and individually with high production costs, being that most producers (44%) manage from 0.5 to 4.9 ha, 30% manage from 5.0 to 49.9 ha, 12% more than 50 ha, 11% of producers drive less than 5 ha, and finally 3% do not own land.^4^ Additionally, through a cluster analysis based on number of cattle, number of milking cows, farm production per day and yield per cow per day, they classified producers in a region of the inter-Andean valleys as: small producers have less than 4 cattle with 2 milking cows, medium producers own 5 to 9 cattle with 2 milking cows and large producer have more than 10 cattle with 7 milking cows,^94^ while^95^ characterized different districts of Puno, which is the region with the largest number of cattle in Peru, and found that the average number of animals per farmer is 23 cattle with 15 milking cows.

There are different production systems in Peru, which vary according to the region where they are mainly located. Three production systems can be distinguished: the extensive system that predominates in the highlands and jungle. In the Amazon region (jungle) of Peru, cattle raising is characterized by the predominance of local breeds adapted to the environmental conditions of the area, which are usually crossed with zebu and European breeds,^96^ while in the highlands, dual-purpose cattle are raised, which live by grazing and are gathered for milking; in both cases, cattle feeding is mainly based on the use of natural pastures.^97^ In the coastal and inter-Andean valleys, the intensive and semi-intensive systems predominate, respectively.^98^

The extensive system is characterized by animals with low productive and reproductive indexes that lack registration, do not have qualified professional for management, producers have access to veterinary services that come from 97% private and 3% from the public sector, which shows the lack of state assistance,^94^ in addition, the labor force is mainly family-based. On dairy farms, it is reported that 62.5% of dairy producers use family labor.^95^

Dairy production in Peru takes place in the country’s three main geographic regions: coast, highlands and jungle. However, the most developed sectors are located on the coast, which is home to 78% of specialized dairy cattle. The dairy basins of Cajamarca, Arequipa and Lima are the most productive, concentrating 60% of total milk production. In addition, the participation of the large industry in milk collection has increased considerably, currently exceeding 50% of national production (FAO, 2009).

Reproductive management and genetic improvement in Peruvian livestock show significant regional differences. On the coast and in specialized dairy basins, breeds such as Holstein, Brown Swiss and Jersey are used in intensive production systems. In contrast, in the highlands, Creole cattle predominate along with Brown Swiss, with limited adoption of artificial insemination. In the jungle, where zebu crossbred cattle prevail, production is dual-purpose (milk and meat), and the use of artificial insemination is minimal.^61^

Livestock feeding in Peru varies considerably by region. On the coast, the main sustenance is based on forage and cultivated pastures, while in the highlands it is supplemented with feed supplements. Livestock farming faces significant health challenges, including zoonotic diseases such as tuberculosis and brucellosis, which continue to affect productivity. The National Agricultural Health Service (SENASA) is in charge of programs aimed at controlling and eradicating these diseases (MINAGRI, 2017).

Annual per capita milk consumption in Peru is 87 kg, which is still below the FAO recommended level of 120 kg per person. Although an increase in meat production has been observed, the country continues to rely heavily on imports to meet domestic demand, underscoring the need to continue improving the productivity and competitiveness of the livestock sector to move toward sustainable development (FAO, 2017).

In 2022, the National Institute for Agrarian Innovation (INIA) achieved several significant milestones in bovine genetic improvement in Peru. First, the birth of “Victoria”, the first Gyr calf obtained through in vitro fertilization (IVF), was achieved, a crucial advance that strengthens the genetic quality and productivity of cattle breeding in the country.^99^ This effort is part of a broader INIA strategy, which includes a project launched in 2022 to improve cattle genetics in six regions, including Cajamarca, Ancash, Ayacucho and Puno. This project seeks to increase milk and beef production through the introduction of improved genes, technical assistance in artificial insemination and distribution of high quality semen straws and embryos.^100^

INIA also presented “Purita”, a heifer of the Girolando breed, developed through reproductive biotechnology as part of the PROMEGNACIONAL project. Purita” was born at the Donoso Agrarian Experimental Station and stands out for its capacity to produce high quality milk, adapting to the climatic conditions of the Amazon and the northern coast of the country.^101^

The lack of adequate infrastructure for livestock breeding, care and reproduction, coupled with limited financial resources, hinders the implementation of modern and sustainable production systems. This limits the ability to make genetic improvements, and make use of genomics, which could increase the productivity and sustainability of livestock production, crucial for economic development and food security in the country. To overcome these obstacles, it is essential to invest in adequate infrastructure and foster access to technologies and knowledge that promote more efficient and sustainable livestock production.

### Awareness and training

Awareness and training in livestock practices are crucial to meet the challenge of bovine genetic improvement in Peru, as they enable farmers to understand and apply advanced techniques that optimize productivity, adaptation to local conditions, environmental sustainability and animal welfare. This training not only improves the efficiency and profitability of livestock production, but also promotes sustainable practices and reduces environmental impacts, thus contributing to a more competitive and environmentally friendly livestock production.,^95^ carried out the characterization of dairy production systems, in which it was determined that the use of a dormant alfalfa variety for cattle feeding reduces the production costs per liter of milk. This study identified that only 29.17% of farmers use artificial insemination with genetically improved bulls, 58.33% only use artificial insemination and 12.5% use natural mating. At the national level, according to,^4^ out of 1753989 producers, only 3.1% use artificial insemination and 4.1% use pedigree sires for cattle improvement.

There is a need for training in the management of genomic technologies, and it is possible that there is a lack of awareness among cattlemen about the benefits of bovine genomics and how they can apply it in their operations. It is reported that only 29% of the producer’s access training on pasture management, genetic improvement, health, feeding, etc. of which 33%, 29% and 13% are small, medium and large producers respectively.^94^ Regularly, since producers express little interest in the talks, which they comment that, in their opinion, are very academic, i.e., not directly related to their agricultural conditions and practices, their participation is motivated by access to individual services (technical assistance, insemination, purchase of medicines at convenient prices, credits, among others).^102^

Effective technology transfer from research to field practice is crucial for bovine genomics to have a real impact on livestock production in Peru. This may involve the creation of extension and training programs for farmers, as well as collaboration with the private sector to facilitate the adoption of genomic technologies. That is why the Ministry of Agrarian Development and Irrigation (MIDAGRI), through the NATIONAL INSTITUTE OF AGRICULTURAL INNOVATION (INIA), has been developing projects in the livestock sector (PROMEGTROPICAL, PROMEGNACIONAL and PROGAN) through which it offers and provides various technical training on genetic improvement of cattle to producers in the country. These are conducted by the multidisciplinary teams that are part of the Experimental Stations at the National level. As well as various courses that complement the empirical training of many of the producers in the area. With the application of these methodologies, it is possible to increase the production of forage and high value cattle feed, as well as to obtain bovine offspring with the capacity to develop quality meat and milk with high indexes of solids and nutrients.^103^

On the other hand, there are also associations that are responsible for working together with producers and implementing personalized and group training, animal health and feeding, many of which are associated with municipal or regional livestock projects. In addition, it is mentioned that technology transfers are complemented with resources that favor the development of these livestock activities, such as: organic conservation of agricultural soil, silvopastoral techniques, production of forage and seeds of high genetic quality, soil analysis for agricultural purposes, organic fertilizer, among others of importance for the activity.^104^

### Costs

Implementing bovine genomics can be costly, both in terms of equipment and genomic analysis. This can be especially challenging for small farmers or those with limited financial resources. The implementations of infrastructure, as well as the development of this item is basically given with the economic budget that the Ministry of Economy and Finance grants to public institutions such as Universities or National Institutes likewise there are other institutions that allow through PROCIENCIA competitive funds, which generate the interest of professionals and/or technicians through economic support. The implementation of facilities with easy access to them at any time of the year since livestock should be located in a site of higher altitude than the surrounding area, to avoid flooding.^88^ The location should be close to supply markets, slaughterhouses and mills, in order to reduce transportation costs for inputs, food, and facilitate access with animals ready for slaughter. Likewise, it should be free of industrial contamination sources and far from landfills and dumps. With sufficient natural air flow, respecting the temperature required by the animals. For this, the land must not be muddy or floodable and with a very good drainage, clean and functional in a continuous way. For extensive livestock raising, the land must have sufficient fodder soil or soil that is native to the area with flora that can be used as feed for the livestock in all its phases, or in the case of pre-fattening or fattening based on pasture. Finally, there must be peripheral fences that allow its delimitation and prevent the entrance of people, animals and vehicles.^105^

For the improvement of the production of milk and dairy products, there is the implementation of milk quality modules, as well as demonstration plants with the aim of obtaining sanitary registration for dairies, and thus generate a Training and Technology Transfer Center, with the implementation of a Demonstrative Cheese Module in strategic locations.^106^ However, it should be considered that there is a limited number of researchers, as well as low interaction with extensionists or technology transfer agents. As well as the low capacity and political interest of regional and local governments (MIDAGRI, INIA, CATIE, 2019).

### Regulations and policies

There may be regulatory or policy barriers that hinder the adoption of genomic technologies in livestock production. The following are some policy regulations that were established in the country:

The Ministry of Agrarian Development and Irrigation - MIDAGRI published Supreme Decree 0010-2022-MIDAGRI, which approves the Sanitary Regulations for the Production and Commercialization of Genetic Material of Production Animals. Official Gazette El Peruano. It covers the provisions for the application of sanitary measures in the production and commercialization of genetic material of production animals, contributing to the epidemiological surveillance, prevention, control and eradication of diseases that affect animals in order to avoid the propagation and dissemination of diseases that affect the sanitary status of the country.

Law N° 27104,^107^ this law establishes: “The general rules applicable to research activities, production, introduction, manipulation, transport, storage, conservation, exchange, commercialization, confined use and release with Living Modified Organism (LMO), under controlled conditions” and excludes: “Activities in human genome, to all types of vaccines applied to human beings, to organisms whose genetic modification is obtained through conventional techniques and traditional methods: in vitro fertilization, conjugation, transduction, transformation or any other natural process; polyploid induction, mutagenesis, formation and utilization of animal hybridoma somatic cells; provided they do not involve the manipulation of recombinant deoxyribonucleic acid (DNA) molecules or the use of LMOs as vector, receptor or parental organisms”.

Law N° 29811^108^ This law establishes: “A moratorium on the entry and production of LMOs in the national territory for a period of 10 years (2011 - 2021), which is a milestone in the policy of conservation of our genetic diversity and capacity building in biosafety. “The purpose of the Moratorium is to strengthen national capacities in terms of human resources, procedures and infrastructure, as well as to generate baselines of the main native and naturalized crops that could be affected by the release of LMOs into the environment. The aim is that, at the end of the moratorium period, the country will be in a position to make responsible decisions and adequately manage the risks related to the entry and use of LMOs, ensuring minimal impacts on biological diversity. The scope of the Moratorium Law covers only LMOs that will be released into the environment as crop or breeding, and excludes LMOs for research under contained use, for food and feed, and pharmacological uses.”

Law N° 31111^109^ This Law establishes that, until December 31, 2035, the moratorium that prevents the entry and production in the national territory of LMOs for cultivation or breeding purposes, including aquatic organisms, to be released into the environment.

Although there are some laws that mention regulations for the production and commercialization of genetic material of livestock and LMOs, there is no consensus between the government and the scientific community.

Several regulations govern the production and commercialization of genetic material in Peru. Supreme Decree 0010-2022-MIDAGRI establishes sanitary regulations for the production and commercialization of genetic material of livestock, ensuring epidemiological surveillance, prevention, control, and eradication of diseases that affect animal health. Additionally, laws such as Law N° 27104, Law N° 29811, and Law N° 31111 regulate the introduction and production of living modified organisms (LMOs), with a moratorium on their use in agriculture and livestock until 2035.

Similar regulatory frameworks exist in other Latin American countries. In Brazil, the National Plan for Livestock Genetic Improvement (PNMGB) has been instrumental in integrating genomic selection into cattle breeding programs, supported by EMBRAPA and private sector partnerships.^110^ Additionally, Brazil’s National Plan for Low-Carbon Agriculture (ABC Plan) and Integrated Crop-Livestock-Forestry (ICLF) policies have successfully incentivized sustainable intensification, pasture rehabilitation, and genetic improvement strategies.^111^ These initiatives have helped Brazil enhance productivity while minimizing environmental impacts.

Likewise, Argentina’s National Genetic Improvement Program for Angus cattle, implemented in 2019, has demonstrated tangible benefits in selection accuracy and genetic progress.^112^ The inclusion of genomic data in breeding value estimations has led to a 4% increase in selection accuracy, providing a strong model for genomic breeding programs.

However, unlike Brazil and Argentina, Peru lacks a centralized genomic breeding policy, limiting the widespread adoption of genomic technologies. The development of a national genomic strategy, similar to those implemented in Brazil and Argentina, could enhance the competitiveness and sustainability of Peruvian livestock. Establishing genomic selection programs and integrating pasture management techniques would allow Peru to improve productivity and ensure the long-term sustainability of cattle breeding while addressing challenges such as climate adaptation and economic viability.

## Opportunities and proposed solutions

Forage quality is a critical factor in reducing enteric methane emissions from dairy cows, a key aspect for the sustainability of livestock production. A study in the Peruvian Andes compared cultivated pastures with native pastures, finding that the former reduced methane emissions per unit of digested organic matter and per unit of energy-corrected milk. This is attributed to the higher nutritional quality of the cultivated pastures, which include more crude protein and metabolizable energy, improving the digestive efficiency of the animals.^113^ Thus, the implementation of improved pastures is presented as an effective strategy to mitigate greenhouse gas emissions in high mountain regions.

Creole cattle, adapted to adverse conditions, not only offer a sustainable option for meat production, but also open the door to the diversification of livestock products in Peru. Promoting organic and grass-fed meat production using Creole cattle can open new markets and improve the competitiveness of the sector. To maximize these benefits, it is essential to develop training and education programs for farmers, as well as to encourage research in genomics applied to these breeds.^63^

In addition, the analysis of carbon footprints associated with different dietary patterns in Peru underscores the importance of promoting sustainable dietary practices to reduce greenhouse gas (GHG) emissions. The average diet in Peru, with a high consumption of animal products, is linked to higher GHG emissions compared to a plant-based diet. There is a positive correlation between socioeconomic status and GHG emissions, indicating that higher income households have a higher dietary carbon footprint. These findings emphasize the need for policies that promote food sustainability and align nutritional policies with environmental objectives, also considering socioeconomic factors in the design of climate change mitigation strategies.^114^

The challenges in adopting genomic technologies in Peruvian livestock farming are not unique to the country. Similar barriers, including infrastructure limitations, financial constraints, and resistance to new technologies, have been observed in other Latin American nations, such as Bolivia and Ecuador, where small-scale farmers dominate the industry and genomic breeding programs remain underdeveloped.^115^^,^^116^ In contrast, countries like Brazil and Argentina have made significant progress in genetic improvement programs, largely due to greater investment in research, stronger institutional support, and collaboration with international genomic initiatives.^112^^,^^117^^,^^118^

Argentina, for example, implemented a national genomic selection program for Angus cattle in 2019, incorporating genomic data into breeding value estimations. This initiative has already led to a 4% increase in selection accuracy, demonstrating the tangible benefits of integrating genomic tools into livestock improvement strategies.^112^ Moreover, ongoing research in Argentina continues to refine genomic selection methodologies, including the use of GBLUP and single-step evaluation models to enhance prediction accuracy.^112^ These advancements serve as a valuable reference for Peru, where large-scale genomic breeding programs have yet to be established. Learning from these experiences could help Peru develop strategies to overcome its own barriers and enhance the adoption of genomic technologies in the livestock sector.

The evolution of genomic technologies has played a transformative role in cattle breeding worldwide, with many countries adopting national genomic selection programs. For instance, Canada, Ireland, and Australia have successfully integrated genomic evaluations into national breeding programs, significantly improving the accuracy of estimated breeding values and increasing genetic gains.^119^^,^^120^ These experiences highlight the importance of strong institutional frameworks, government incentives, and public-private collaboration in accelerating the adoption of genomic selection. In Peru, adapting similar policies, such as funding genomic research and establishing national reference genomic databases, could provide the necessary infrastructure for genomic breeding programs. Additionally, aligning local strategies with international initiatives, such as the Bovine Pangenome Consortium,^71^ would enhance Peru’s integration into the global genomic research network, facilitate access to advanced technologies, and promote sustainable livestock development.

## Conclusions and recommendations

The implementation of bovine genomics in Peru is emerging as a crucial tool to address the challenges of sustainability and productivity in livestock farming, especially in a context of environmental and socioeconomic constraints. Creole cattle, with their adaptability to adverse conditions, offer an invaluable genetic base for improving meat and milk production; while promoting the conservation of local biodiversity, genomic analysis of these creole cattle is essential for crossbreeding, as Peru also has specialized breeds of genetic material. However, it is essential to strengthen the infrastructure and training of producers to facilitate the adoption of these technologies. In addition, a public policy framework is needed to promote research and development of applied biotechnologies, ensuring a transition to more sustainable and efficient practices in the Peruvian livestock sector. Inter-institutional cooperation and technical and financial support are essential to enhance the impact of genomics on improving the quality and competitiveness of livestock production in the country.

## References

[1] Ministerio de Economía y Finanzas (MEF). Memorial institucional 2019; 2020. https://www.smv.gob.pe/ConsultasP8/temp/Memoria 2019 - 1.pdf.

[2] MINAGRI. Plan Nacional de Desarrollo Ganadero 2017-2027. 2017b. https://www.planificacion.gob.ec/wp-content/uploads/downloads/2017/10/PNBV-26-OCT-FINAL_0K.compressed1.pdf.

[3] Instituto Nacional de Estadística e Informática (INEI). *Base de Datos Del Sistema Nacional de Consulta Del IV CENAGRO (Censo Nacional Agropecuario)*. Lima, Perú: INEI; 2012.

[4] INEI. *IV Censo Nacional Agropecuario. Resultados Definitivos.* Lima, Peru: INEI; 2012. http://proyectos.inei.gob.pe/web/DocumentosPublicos/ResultadosFinalesIVCENAGRO.pdf

[5] Rosemberg M. Ganaderia bovina en el Perú. *Agronoticias*. 2017; 432:43–47. https://www.inei.gob.pe/media/inei_en_los_medios/Agronoticias-43-44-45-46-47.pdf

[6] Fuentes E, Gómez C, Pizarro D, et al. A review of silvopastoral systems in the Peruvian Amazon region. *Trop grassl-Forrajes Trop*. 2022;10(2):78–88. (10)78-88

[7] Wiggans GR, Cole JB, Hubbard SM, Sonstegard TS. Genomic selection in dairy cattle: The USDA experience. *Annu Rev Anim Biosci*. 2017;5(1):309–327. 27860491 10.1146/annurev-animal-021815-111422

[8] FAO. Artificial Insemination and Breeding Development Programme: Mission to Peru. Rome, Italy: Food and Agriculture Organization of the United Nations; 1978.

[9] Gloria SA. Boletín Técnico de Gloria S.A.: “El Poronguito. Lima, Perú: Gloria S.A; 1986.

[10] Cabrera P. BNS de la UNALM Ofrece Semen Bovino a Precios Bajos para Ganaderos Peruanos. (Entrevista). 2014. Lima, Peru: Perulactea; https://perulactea.com/bns-de-la-unalm-ofrece-semen-bovino-a-precios-bajos-para-ganaderos-peruanos/.

[11] United Nation Statistics Division. UNDATA UNSD Statistical Databases. New York, USA: United Nations; 2014. http://data.un.org/Data.aspx?q=semen&d=ComTrade&f=_l1Code%3a6%3bcmdCode%3a051110.

[12] Montoya Barrera BMM. Parentesco y Consanguinidad de Bovinos Simmental y Fleckvieh Inscritos en Los Registros Genealógicos Zootécnicos Del Perú (1982–2018) [Tesis de licenciatura]. Universidad Nacional Agraria La Molina; 2018.

[13] Barrón JA. Fleckvieh en la Universidad Nacional Agraria La Molina. (Entrevista). Lima, Peru: Universidad Nacional Agraria La Molina, Programa de Investigación y Proyección Social en Mejoramiento Animal; 2016.

[14] Pahuara LE, Naveros M. Producción in vitro de embriones bovinos (*Bos taurus*) en dos medios de cultivo. *Spermova*. 2014;21(2):54–57.

[15] UNTRM. Patente de investigación de UNTRM es admitida en XV Concurso Nacional de Invenciones; 2016. https://www.untrm.edu.pe/portal/es/noticias/1100-patente-de-investigacion-de-untrm-es-admitida-en-xv-concurso-nacional-de-invenciones.html?highlight=WyJjbG9uYWNpXHUwMGYzbiIsImNsb25hY2lvbiJd.

[16] Andina. 2020. ¡Proeza científica! Investigadores de Amazonas obtienen dos clones de vaca alemana. Andina. https://andina.pe/agencia/noticia-proeza-cientifica-investigadores-amazonas-obtienen-dos-clones-vaca-alemana-825983.aspx.

[17] UNTRM. UNTRM logra producción de descendencia de clones bovinos. 2023. https://www.untrm.edu.pe/portal/es/noticias/3724-untrm-logra-produccion-de-descendencia-de-clones-bovinos.html.

[18] Frankham R, Ballou JD, Briscoe DA. Introduction to conservation genetics. 2nd ed. Cambridge, UK: Cambridge University Press; 2002.

[19] Sonesson AK, Woolliams JA, Meuwissen THE. Genomic selection requires genomic control of inbreeding. *Genet Sel Evol*. 2012;44(1):27. 22898324 10.1186/1297-9686-44-27 PMC3522025

[20] Lee M. DNA markers and plant breeding programs. *Adv Agron*. 1995;55:265–344.

[21] Georges M, Lathrop M, Bouquet Y, et al. Linkage relationships among 20 genetic markers in cattle: Evidence for linkage between two pairs of blood group systems: B-Z and S-F/V, respectively. *Anim Genet*. 1990;21(2):95–105. 2386316 10.1111/j.1365-2052.1990.tb03213.x

[22] Weller JI, Ezra E, Ron M. Invited review: A perspective on the future of genomic selection in dairy cattle. *J Dairy Sci*. 2017;100(11):8633–8644. 28843692 10.3168/jds.2017-12879

[23] Jiang L, Wang C, Zhou X, Liu J. Simulation-based evaluation of genomic selection strategies in beef cattle breeding programs. *Front Genet*. 2023;14:1083106. 37007975 10.3389/fgene.2023.1083106 PMC10064214

[24] Zhang H, Du H, Wang Y, Zhang Q. Application of genomic selection in beef cattle disease prevention. *Animals*. 2025;15(2):277. 39858277 10.3390/ani15020277 PMC11759163

[25] Kadarmideen HN. Genomics to systems biology in animal and veterinary sciences: Progress, lessons and opportunities. *Livest Sci*. 2014;166:232–248.

[26] Boichard D, Chung H, Dassonneville R, et al. Design of a bovine low-density SNP array optimized for imputation. *PLoS One*. 2012;7(3):e34130. 22470530 10.1371/journal.pone.0034130 PMC3314603

[27] Elsik CG, Tellam RL, Worley KC, et al. The genome sequence of taurine cattle: A window to ruminant biology and evolution. *Science*. 2009;324(5926):522–528. 19390049 10.1126/science.1169588 PMC2943200

[28] Kõks S, Lilleoja R, Reimann E, Salumets A, Reemann P, Jaakma Ü. Sequencing and annotated analysis of the Holstein cow genome. *Mamm Genome*. 2013;24(7-8):309–321. 23893136 10.1007/s00335-013-9464-0

[29] Stafuzza NB, Zerlotini A, Lobo FP, et al. Single nucleotide variants and InDels identified from whole-genome re-sequencing of Guzerat, Gyr, Girolando and Holstein cattle breeds. *PLoS One*. 2017;12(3):e0173954. 28323836 10.1371/journal.pone.0173954 PMC5360315

[30] Carthy TR, McCarthy J, Berry DP. A mating advice system in dairy cattle incorporating genomic information. *J Dairy Sci*. 2019;102(9):8210–8220. 31229287 10.3168/jds.2019-16283

[31] Robertson JS. Polymerase chain reaction (PCR) and sequencing. *Dev Biol Stand*. 1994;83:81–85. 7883102

[32] Cañón J, Alexandrino P, Bessa I, et al. Genetic diversity measures of local European beef cattle breeds for conservation purposes. *Genet Sel Evol*. 2001;33(3):311–332. 11403750 10.1186/1297-9686-33-3-311 PMC2705410

[33] Delgado JV, Martínez AM, Acosta A, et al. Genetic characterization of Latin-American Creole cattle using microsatellite markers. *Anim Genet*. 2012;43(1):2–10. 10.1111/j.1365-2052.2011.02207.x 22221019

[34] Eusebi PG, Cortés O, Dunner S, Cañón J. Genetic diversity of the Mexican Lidia bovine breed and its divergence from the Spanish population. *J Anim Breed Genet*. 2017;134(4):332–339. 28033673 10.1111/jbg.12251

[35] Ginja C, Gama LT, Cortés O, et al. The genetic ancestry of American Creole cattle inferred from uniparental and autosomal genetic markers. *Sci Rep*. 2019;9(1):11486. 31391486 10.1038/s41598-019-47636-0 PMC6685949

[36] Lenstra JA, Groeneveld LF, Eding H, et al. Molecular tools and analytical approaches for the characterization of farm animal genetic diversity. *Anim Genet*. 2012;43(5):483–502. 22497351 10.1111/j.1365-2052.2011.02309.x

[37] Fernando RL, Grossman M. Marker assisted selection using best linear unbiased prediction. *Genet Sel Evol*. 1989;21(4):467.

[38] Sahana G, Guldbrandtsen B, Thomsen B, Lund MS. Confirmation and fine-mapping of clinical mastitis and somatic cell score QTL in Nordic Holstein cattle. *Anim Genet*. 2013;44(6):620–626. 23647142 10.1111/age.12053

[39] Sternstein I, Reissmann M, Maj D, Bieniek J, Brockmann GA. A comprehensive linkage map and QTL map for carcass traits in a cross between Giant Grey and New Zealand White rabbits. *BMC Genet*. 2015;16(1):16. 25887754 10.1186/s12863-015-0168-1 PMC4330979

[40] Yoo CK, Park HB, Lee JB, et al. QTL analysis of body weight and carcass body length traits in an F2 intercross between Landrace and Korean native pigs. *Anim Genet*. 2014;45(4):589–592. 24797173 10.1111/age.12166

[41] Toro MA, Fernández J, Caballero A. Molecular characterization of breeds and its use in conservation. *Livest Sci*. 2009;120(3):174–195.

[42] Pérez-Enciso M, Rincón JC, Legarra A. Sequence- vs. chip-assisted genomic selection: Accurate biological information is advised. *Genet Sel Evol*. 2015;47(1):43. 25956961 10.1186/s12711-015-0117-5 PMC4424891

[43] Mu Y, Qi W, Zhang T, Zhang J, Mao S. Multi-omics Analysis revealed coordinated responses of rumen microbiome and epithelium to high-grain-induced subacute rumen acidosis in lactating dairy cows. *mSystems*. 2022;7(1):e0149021. 35076273 10.1128/msystems.01490-21 PMC8788321

[44] Wang Y, Li J, Lu D, et al. Integrated proteome and phosphoproteome analysis of interscapular brown adipose and subcutaneous white adipose tissues upon high fat diet feeding in mouse. *J Proteomics*. 2022;255:104500. 35101640 10.1016/j.jprot.2022.104500

[45] Matukumalli LK, Lawley CT, Schnabel RD, et al. Development and characterization of a high density SNP genotyping assay for cattle. *PLoS One*. 2009;4(4):e5350. 19390634 10.1371/journal.pone.0005350 PMC2669730

[46] Bovo S, Ribani A, Muñoz M, et al. Whole-genome sequencing of European autochthonous and commercial pig breeds allows the detection of signatures of selection for adaptation of genetic resources to different breeding and production systems. *Genet Sel Evol*. 2020;52(1):33. 32591011 10.1186/s12711-020-00553-7 PMC7318759

[47] Wu Y, Zeng J, Zhang F, et al. Integrative analysis of omics summary data reveals putative mechanisms underlying complex traits. *Nat Commun*. 2018;9(1):918. 29500431 10.1038/s41467-018-03371-0 PMC5834629

[48] Ng B, Casazza W, Kim NH, et al. Cascading epigenomic analysis for identifying disease genes from the regulatory landscape of GWAS variants. *PLoS Genet*. 2021;17(11):e1009918. 34807913 10.1371/journal.pgen.1009918 PMC8648125

[49] Chang LY, Toghiani S, Aggrey SE, Rekaya R. Increasing accuracy of genomic selection in presence of high density marker panels through the prioritization of relevant polymorphisms. *BMC Genet*. 2019;20(1):21. 30795734 10.1186/s12863-019-0720-5 PMC6387489

[50] Romero R, Espinoza J, Gotsch F, et al. The use of high-dimensional biology (genomics, transcriptomics, proteomics, and metabolomics) to understand the preterm parturition syndrome. *BJOG*. 2006;113 Suppl 3(Suppl 3):118–135. 17206980 10.1111/j.1471-0528.2006.01150.x PMC7062297

[51] Krassowski M, Das V, Sahu SK, Misra BB. State of the field in multi-omics research: from computational needs to data mining and sharing. *Front Genet*. 2020;11:610798. 33362867 10.3389/fgene.2020.610798 PMC7758509

[52] Estrada R, Corredor FA, Figueroa D, et al. Reference-guided draft genome assembly, annotation and SSR mining data of the Peruvian creole cattle (Bos taurus). *Data (Basel)*. 2022;7(11):155.

[53] Corredor F-A, Figueroa D, Estrada R, et al. Genetic diversity and population structure of a Peruvian cattle herd using SNP data. *Front Genet*. 2023;14:1073843. 36968592 10.3389/fgene.2023.1073843 PMC10036791

[54] Arbizu CI, Ferro-Mauricio RD, Chávez-Galarza JC, et al. The complete mitochondrial genome of a neglected breed, the Peruvian Creole Cattle (Bos taurus), and its phylogenetic analysis. *Data (Basel)*. 2022;7(6):76.

[55] Estrada R, Figueroa D, Romero Y, et al. Complete Mitogenome of “Pumpo” (Bos taurus), a top bull from a Peruvian genetic nucleus, and its phylogenetic analysis. *Curr Issues Mol Biol*. 2024;46(6):5352–5363. 38920992 10.3390/cimb46060320 PMC11201737

[56] Herath M, Hosie S, Bornstein JC, Franks AE, Hill-Yardin EL. The role of the gastrointestinal mucus system in intestinal homeostasis: Implications for neurological disorders. *Front Cell Infect Microbiol*. 2020;10:248. 32547962 10.3389/fcimb.2020.00248 PMC7270209

[57] Cryan JF, Dinan TG. Mind-altering microorganisms: the impact of the gut microbiota on brain and behaviour. *Nat Rev Neurosci*. 2012;13(10):701–712. 22968153 10.1038/nrn3346

[58] Vuong HE, Yano JM, Fung TC, Hsiao EY. The microbiome and host behavior. *Annu Rev Neurosci*. 2017;40(1):21–49. 28301775 10.1146/annurev-neuro-072116-031347 PMC6661159

[59] MINAG. Vacunos de leche: situación actual. 2014. http://www.minag.gob.pe/portal/sector-agrario/pecuaria/situacion-de-las-actividadesde-crianza-y-produccion/vacunos-de-leche.

[60] Eslava Parra P. Impacto Económico y Social Del Uso de Semen Sexado Nacional en la Ganadería Bovina Del Perú [Tesis de licenciatura]. Universidad Nacional Agraria La Molina; 2014.

[61] MINAG. *Plan Ganadero Nacional Para el Desarrollo Ganadero 2006 al 2015*. Lima, Perú: MINAG; 2006. p. 73.

[62] MIDAGRI. MINAGRI desarrolla con éxito mejoramiento genético de ganado vacuno. 2018. https://www.midagri.gob.pe/portal/noticias-anteriores/notas-2018/21108-minagri-desarrolla-con-exito-mejoramiento-genetico-de-ganado-vacuno.

[63] Márquez-Godoy JN, Álvarez-Holguín A, Morales-Nieto CR, Corrales-Lerma R, García-Galicia IA, Rodríguez-Almeida FA. Criollo cattle breeds as a potential alternative for sustainable and healthy beef production in America. *Rangeland Ecol Manage*. 2024;96:83–93.

[64] Alfaro-Astorima MI, Ormachea-Sánchez HH, Alvarado-Malca AE. Ovarian follicular dynamics of a creole cattle under grazing conditions in high Andean areas of Peru. *Sci Agropecu*. 2020;11(4):621–628.

[65] Arias-Pacheco C, Lucas JR, Rodríguez A, Córdoba D, Lux-Hoppe EG. Economic impact of the liver condemnation of cattle infected with Fasciola hepatica in the Peruvian Andes. *Trop Anim Health Prod*. 2020;52(4):1927–1932. 31965412 10.1007/s11250-020-02211-y

[66] Yalta-Macedo CE, Veli EA, Díaz GR, Vallejo-Trujillo A. Paternal ancestry of Peruvian creole cattle inferred from Y-chromosome analysis. *Livest Sci*. 2021;244:104376.

[67] Figueroa D, Saldaña CL, Corredor F, et al. Genetic diversity and structure of creole cattle (Bos taurus) from southern Peruvian Highlands. Plant & Animal Genome Conference 2024 (PAG 31); Jan 12–17; San Diego, CA, USA. San Diego, CA: PAG; 2024.

[68] Delgado C A, García B C, Allcahuamán M D, Aguilar G C, Estrada V P, Vega A H. Caracterización fenotípica del ganado criollo en el Parque nacional Huascarán – Ancash. *Rev Investig Vet Perú*. 2019;30(3):1143–1149.

[69] Zimin AV, Delcher AL, Florea L, et al. A whole-genome assembly of the domestic cow, Bos taurus. *Genome Biol*. 2009;10(4):R42. 19393038 10.1186/gb-2009-10-4-r42 PMC2688933

[70] Ghavi Hossein-Zadeh N. An overview of recent technological developments in bovine genomics. *Vet Anim Sci*. 2024;25:100382. 39166173 10.1016/j.vas.2024.100382 PMC11334705

[71] Smith TPL, Bickhart DM, Boichard D, et al. The Bovine Pangenome Consortium: democratizing production and accessibility of genome assemblies for global cattle breeds and other bovine species. *Genome Biol*. 2023;24(1):139. 37337218 10.1186/s13059-023-02975-0 PMC10278262

[72] Espinosa E, Bautista R, Fernandez I, Larrosa R, Zapata EL, Plata O. Comparing assembly strategies for third-generation sequencing technologies across different genomes. *Genomics*. 2023;115(5):110700. 37598732 10.1016/j.ygeno.2023.110700

[73] Armstrong S, Bhide P, Jordan V, Pacey A, Marjoribanks J, Farquhar C. Time-lapse systems for embryo incubation and assessment in assisted reproduction. *Cochrane Database Syst Rev*. 2019; 2019(5):CD011320. 10.1002/14651858.CD011320.pub4 PMC6539473 31140578

[74] Romar R, Cánovas S, Matás C, Gadea J, Coy P. Pig in vitro fertilization: Where are we and where do we go? *Theriogenology*. 2019;137:113–121. 31182223 10.1016/j.theriogenology.2019.05.045

[75] Hillyear LM, Zak LJ, Beckitt T, Griffin DK, Harvey SC, Harvey KE. Morphokinetic profiling suggests that rapid first cleavage division accurately predicts the chances of blastulation in pig in vitro produced embryos. *Animals*. 2024;14(5):783. 38473168 10.3390/ani14050783 PMC10930457

[76] Pay Bosch E, Bori L, Beltran A, Naranjo V, Meseguer M. P–141 Artificial intelligence system for the automation of the blastocyst morphology evaluation in GERI Time-lapse Incubator. *Human Reproduction*. 2021;36(Supplement_1):deab130–140.

[77] Apter S, Ebner T, Freour T, et al. Good practice recommendations for the use of time-lapse technology. *Hum Reprod Open*. 2020;2020(2):hoaa008. 32206731 10.1093/hropen/hoaa008 PMC7081060

[78] Singh AK, Kumar A, Bisla A. Computer-assisted sperm analysis (CASA) in veterinary science: A review. *Indian J of Anim Sci*. 2021;91(6):419–429.

[79] Pichardo-Matamoros D, Sevilla F, Salazar JE, et al. Exploration of semen quality analyzed by CASA mot systems of brahman bulls infected with BLV and BHV 1. *Sci Rep*. 2023;13(1):18659. 37907654 10.1038/s41598-023-45981-9 PMC10618460

[80] MIDAGRI. *Anuario Estadístico: Producción Ganadera y Avícola 2020*. Lima, Perú: MIDAGRI; 2021.

[81] Diaz-Quevedo C, Frias H, Cahuana GM, Tapia-Limonchi R, Chenet SM, Tejedo JR. High prevalence and risk factors of fascioliasis in cattle in Amazonas, Peru. *Parasitol Int*. 2021;85:102428. 34329752 10.1016/j.parint.2021.102428

[82] Gómez CA, Fernández M, Franco N, Cueva R. Effect of two formulations of recombinant bovine somatotropin on milk production and body condition of cattle under intensive management in Peru. *Trop Anim Health Prod*. 2022;54(2):96. 35138491 10.1007/s11250-021-03036-z PMC8827379

[83] Vásquez R A, García S MEC, Sessarego D E, Chagray A N. Modelación de la curva de lactación en vacas Holstein de un establo en el valle de Huaura, Perú. *Rev Investig Vet Perú*. 2021;32(1):e19488.

[84] Torres Jara de García GP, Durand-Chávez LM, Quispe-Ccasa HA, et al. Sustainability of livestock farms: The case of the district of Moyobamba, Peru. *Heliyon*. 2023;9(2):e13153. 36755598 10.1016/j.heliyon.2023.e13153 PMC9900507

[85] Redding LE, Barg FK, Smith G, Galligan DT, Levy MZ, Hennessy S. The role of veterinarians and feed-store vendors in the prescription and use of antibiotics on small dairy farms in rural Peru. *J Dairy Sci*. 2013;96(11):7349–7354. 24054290 10.3168/jds.2013-7045 PMC4197058

[86] Lennox E, Gowdy J. Ecosystem governance in a highland village in Peru: Facing the challenges of globalization and climate change. *Ecosyst Serv*. 2014;10:155–163.

[87] Universidad Nacional Agraria La Molina. Productividad Lechera. 2023. http://www.lamolina.edu.pe/mejoramientoanimal/ProductividadLechera.htm.

[88] MINAGRI. Diagnóstico de Crianzas Priorizadas para el Plan Ganadero. 2017a. https://repositorio.minagri.gob.pe/jspui/bitstream/MINAGRI/328/1/plan-ganadero-2017-2021.pdf.

[89] Martínez-López B, Ivorra B, Fernández-Carrión E, et al. A multi-analysis approach for space–time and economic evaluation of risks related with livestock diseases: The example of FMD in Peru. *Prev Vet Med*. 2014;114(1):47–63. 24485278 10.1016/j.prevetmed.2014.01.013

[90] Rodríguez-Campos LA. Clasificación de toros lecheros mediante análisis de factores y análisis de conglomerados. *Nut Anim Trop*. 2019;13(2):1–19.

[91] Gilardino A, Quispe I, Pacheco M, Bartl K. Comparison of different methods for consideration of multifunctionality of Peruvian dairy cattle in Life Cycle Assessment. *Livest Sci*. 2020;240:104151.

[92] Rojas Briceño N, Barboza Castillo E, Gamarra Torres O, et al. Morphometric prioritization, fluvial classification, and hydrogeomorphological quality in high andean livestock micro-watersheds in Northern Peru. *IJGI*. 2020;9(5):305.

[93] Trinidad SEL, Bravo CB, Narvasta SF, et al. Seroprevalence of reproductive and infectious diseases in cattle: the case of Madre de Dios in the Peruvian southeastern tropics. *Am J Vet Res*. 2024;85(4):1–8. 38335721 10.2460/ajvr.23.08.0177

[94] Gamboa C, Mercado W. Comercialización De La Leche En La Provincia De Concepción, Valle Del Mantaro, Junín - Perú. *An Cient UNA*. 2015;76(2):225.

[95] Rivera R, Vargas J, Gomez C. Characterization of milk production systems using dormant alfalfa (Medicago sativa L.). *Livestock Research for Rural Development*. 2016;28(9):152. http://www.lrrd.org/lrrd28/9/rive28152.html

[96] Sánchez Gamarra J, Almeyda Matias J, Isique Huaroma J. Caracterización de los sistemas de producción de vacunos, para el desarrollo ganadero en el distrito de Oxapampa – Pasco. *An Cient UNA*. 2019;80(2):594.

[97] Pando G, Peruano D. Manejo y alimentación del ganado vacuno de doble propósito en sierra. 2010. https://repositorio.inia.gob.pe/bitstream/20.500.12955/164/1/Alimentacion_ganado_vacuno_2010.pdf.

[98] MIDAGRI. Sistemas de Producción. n.d. Retrieved July 22, 2024, from https://www.midagri.gob.pe/portal/40-sector-agrario/situacion-de-las-actividades-de-crianza-y-producci/303-vacunos-de-leche?start=17.

[99] AgroPerú. Nació la primera ternera Gyr por fecundación in vitro en Huaral. 2022. https://www.agroperu.pe/nacio-la-primera-ternera-gyr-por-fecundacion-in-vitro-en-huaral/.

[100] RCR. INIA inicia proyecto de mejoramiento genético de ganado para que puedan producir más leche y carne en seis regiones. 2022. https://www.rcrperu.com/inia-inicia-proyecto-de-mejoramiento-genetico-de-ganado-para-que-puedan-producir-mas-leche-y-carne-en-seis-regiones/.

[101] Gob. INIA pone a disposición a PURITA, nueva ternera de raza Girolando con alta calidad genética. 2022. Retrieved from https://www.gob.pe/institucion/inia/noticias/576136-inia-pone-a-disposicion-a-purita-nueva-ternera-de-raza-girolando-con-alta-calidad-genetica.

[102] Faure G, Huamanyauri Méndez K, Salazar I, Gómez C, De Nys E, Dulcire M. La privatización del asesoramiento agrícola: Consecuencias para los productores lecheros del valle del Mantaro, Perú. *Cuad Des Rural*. 2015;12(76):1.

[103] INIA. Gobierno impulsa el desarrollo ganadero del país con la reproducción de 23 mil crías de bovinos por inseminación artificial. 2024. https://www.gob.pe/institucion/inia/noticias/972098-gobierno-impulsa-el-desarrollo-ganadero-del-pais-con-la-reproduccion-de-23-mil-crias-de-bovinos-por-inseminacion-artificial.

[104] Gobierno Regional San Martín. Proyecto Ganadero replica paquetes tecnológicos en productores de San Martín. 2022. https://www.gob.pe/institucion/regionsanmartin/noticias/590697-proyecto-ganadero-replica-paquetes-tecnologicos-en-productores-de-san-martin.

[105] SENASA. Guía de Buenas Prácticas en la producción de Bovinos de Carne. n.d. https://cdn.www.gob.pe/uploads/document/file/1129274/Guía-BPPBovinos.pdf.pdf. Accessed July 14, 2024.

[106] Caceres J. Mejoramiento de los servicios de la producción bovina de leche en la provincia de Lucanas región Ayacucho. 2017. https://www.google.com/url?sa=i&url=https%3A//ofi5.mef.gob.pe/invierte/general/downloadArchivo%3Ftipo%3DSNIP%26idArchivo%3D3264_GRAYAICTF_201765_171244.pdf&psig=AOvVaw3sN7d1wpsWHG2gfUU3gBar&ust=1723530058817000&source=images&cd=vfe&opi=89978449&ved=0CAQQn5wMahcKEwjouPKq6O6HAxUAAAAAHQAAAAAQBA.

[107] Consejo De Ministros De La Republica Del Peru. Ley 27104. 2017. https://cdn.www.gob.pe/uploads/document/file/12803/Ley-N_-27104.pdf?v=1530656645.

[108] Consejo De Ministros De La Republica Del Peru. Ley 29811. 2012. https://www.minam.gob.pe/wp-content/uploads/2017/04/Ley-N°-29811.pdf.

[109] Consejo De Ministros De La Republica Del Peru. Ley 31111. 2021. https://busquedas.elperuano.pe/dispositivo/NL/1917468-1.

[110] Cunha Alves AA, Azevedo VCR. Embrapa network for Brazilian plant genetic resources conservation. *Biopreserv Biobank*. 2018;16(5):350–360. 30325669 10.1089/bio.2018.0044 PMC6204567

[111] Molossi L, Hoshide AK, de Abreu DC, de Oliveira RA. Agricultural support and public policies improving sustainability in Brazil’s beef industry. *Sustainability*. 2023;15(6):4801.

[112] Curutchet AR, Fragomeni BO, Guitou H, Monti A. 141 exploring alternative approaches for the national argentine angus cattle genomic evaluation program. *J Animal Sci*. 2023;101(Supplement_3):38–39.

[113] Alvarado-Bolovich V, Medrano J, Haro J, Castro-Montoya J, Dickhoefer U, Gómez C. Enteric methane emissions from lactating dairy cows grazing cultivated and native pastures in the high Andes of Peru. *Livest Sci*. 2021;243:104385.

[114] Vázquez-Rowe I, Larrea-Gallegos G, Villanueva-Rey P, Gilardino A. Climate change mitigation opportunities based on carbon footprint estimates of dietary patterns in Peru. *PLoS One*. 2017;12(11):e0188182. 29145461 10.1371/journal.pone.0188182 PMC5690589

[115] Encalada YAJ, Villacorta PAR. Tendencias Tecnológicas con IoT en la Ganadería 4.0 Aplicables en Ecuador. *Ciencia Latina Revista Científica Multidisciplinar*. 2024;8(6):5947–5974.

[116] Bottani G. Bolivian Creole Cattle: population Structure, Genetic Diversity and Management Practices [Doctoral thesis]. Uppsala, Sweden: Swedish University of Agricultural Sciences; 2020.

[117] Simões MR, Leal JJ, Minho AP, et al. Breeding objectives of Brangus cattle in Brazil. *J Anim Breed Genet*. 2020;137(2):177–188. 31179593 10.1111/jbg.12415

[118] Malafaia GC, de Vargas Mores G, Casagranda YG, Barcellos JOJ, Costa FP. The Brazilian beef cattle supply chain in the next decades. *Livest Sci*. 2021;253:104704.

[119] Misztal I, Lourenco D, Legarra A. Current status of genomic evaluation. *J Anim Sci*. 2020;98(4):skaa101. 32267923 10.1093/jas/skaa101 PMC7183352

[120] Banks R. Evolution of genetics organisations’ strategies through the implementation of genomic selection: Learnings and prospects. *Agriculture*. 2022;12(10):1524.

